# Emotional intelligence and childbearing affective reward expectation among female healthcare workers: the chain mediating roles of family emotional labor strategies and emotional exhaustion—evidence from Anhui Province, China

**DOI:** 10.3389/fpubh.2026.1850042

**Published:** 2026-09-11

**Authors:** Xiao Chang, Jin Chen, Jing Wang, Guiping Gong, Panpan Hu, Haifeng Zhang

**Affiliations:** 1 Department of Neurology, Anhui Medical University, Affiliated Hospital 1, Hefei, China; 2 MOE Key Laboratory of Population Health Across Life Cycle, Anhui Medical University, Hefei, China; 3 School of Health Management, Anhui Medical University, Hefei, China

**Keywords:** childbearing affective reward expectation, emotional exhaustion, emotional intelligence, exploratory study, family emotional labor

## Abstract

**Objective:**

To investigate the relationship between emotional intelligence and childbearing affective reward expectation, and analyze the potential roles of family emotional labor strategies and emotional exhaustion in this relationship.

**Methods:**

A two-stage questionnaire survey using the emotional intelligence scale, family emotional labor scale, and emotional exhaustion scale was conducted among 450 female healthcare workers from three affiliated hospitals of a medical college in Anhui Province, located in Hefei, Suzhou, and Fuyang.

**Conclusion:**

This study confirms a positive correlation between emotional intelligence and childbearing affective reward expectation among female healthcare workers of childbearing age and reveals the mediating roles of family emotional labor strategies and emotional exhaustion in this relationship. The findings have theoretical value and practical significance for deepening the understanding of the psychological roots of low fertility intentions and designing fertility support policies that go beyond economic incentives to address emotional needs.

## 1 Introduction

According to national statistics, China recorded 7.92 million births and 11.31 million deaths in 2025, marking the fourth consecutive year of negative population growth. China’s demographic development faces the long-term challenge of a sustained decline in the total fertility rate. Previous studies have mainly focused on the effects of economic factors such as education and housing (1–3), and institutional constraints, such as maternity allowances and childcare services, on fertility (4, 5). However, even under similar economic conditions, significant differences exist in individual fertility intentions, suggesting that psychological and emotional processes play a crucial role in fertility decision-making (6). The decision to have children is not merely a rational calculation process but also a psychological process accompanied by emotional experiences and expectations. Individuals subconsciously evaluate the emotional rewards that childbearing may bring, thereby forming childbearing affective reward expectation. Such expectations influence the perceived attractiveness and priority of childbearing for individuals.

Among the many psychological factors that may influence childbearing affective reward expectation, emotional intelligence is an important individual psychological resource (7), whose role warrants further investigation. Emotional intelligence represents an individual’s ability to perceive, understand, use, and manage emotions and is a key resource for coping with stress, maintaining relationships, and enhancing wellbeing (8). Family emotional labor, which involves regulating and managing emotions to maintain family harmony, provides a context in which the role of emotional intelligence may operate (9). Women are often expected to perform more family emotional labor. Long-term emotional masking may lead to emotional exhaustion (10) and undermine confidence in childbearing and family life. Research on the influence of emotional labor on fertility remains limited. Through qualitative research, Shen Yang identified emotional labor as a factor that may inhibit fertility intentions (11). Using posts by young mothers in the “emotional tree hole” discussion area of an online mother-and-infant community, Zhou Wei examined family emotional labor among young mothers in China and suggested that surface acting may reduce young couples’ intentions to marry and have children (12). Qualitative studies suggest that emotional intelligence may affect fertility behavior, but the mechanisms underlying this relationship remain understudied. Therefore, this study integrates conservation of resources theory and the cognitive assessment perspective to explore the relationship between emotional intelligence and childbearing affective reward expectation and the potential mediating roles of family emotional labor strategies and emotional exhaustion in this relationship.

### 1.1 Emotional intelligence and childbearing affective reward expectation

Conservation of resources theory posits that individuals strive to acquire, retain, and protect valued resources. Emotional intelligence (EI) is an important psychological resource that enables individuals to manage emotional fluctuations more effectively, recover from challenges, and maintain positive emotional states (13). When raising children, individuals with high emotional intelligence may have greater emotional resources and manage them more effectively (14). According to the cognitive assessment theory, individuals with high emotional intelligence are more likely to appraise fertility (what fertility means) as a challenge that can bring about emotional connection, a sense of meaning, and personal growth rather than a threat. During secondary assessment, greater confidence in their ability to manage emotions may lead them to expect that they can meet the emotional needs of parenting, thereby increasing childbearing affective reward expectation (15). Based on this, we propose Hypothesis 1:

*H1*: Emotional intelligence is positively associated with childbearing affective reward expectation.

### 1.2 The mediating role of emotional exhaustion

Emotional exhaustion is a state resulting from the prolonged depletion of emotional resources and is characterized by emotional fatigue, numbness, and low efficacy (16). As a protective resource, high emotional intelligence can help prevent and alleviate emotional exhaustion (17). When faced with stress, individuals with high emotional intelligence can more readily identify signs of resource depletion and adopt effective strategies to maintain their emotional resources. Emotional exhaustion can distort an individual’s cognitive assessment process. When an individual is emotionally exhausted, their cognitive bandwidth narrows, making it difficult to form positive long-term childbearing affective reward expectation. This may amplify short-term emotional costs, thereby reducing childbearing affective reward expectation. Therefore, emotional intelligence may maintain positive childbearing affective reward expectation by reducing the risk of emotional exhaustion. Based on this, we propose Hypothesis 2:

*H2*: Emotional exhaustion mediates the relationship between emotional intelligence and childbearing affective reward expectation.

### 1.3 The chain-mediated role of family emotional labor strategies and emotional exhaustion

Deep acting and surface acting both involve resource loss. The conservation of resources theory holds that individuals have limited resources. Excessive emotional labor inevitably depletes limited emotional resources. Existing research also suggests that both strategies can lead to fatigue (18). In the family setting, sustained surface acting leads to continuous emotional dissonance, making individuals feel inauthentic and threatening their self-identity and wellbeing. Compared with surface acting, deep acting achieves consistent emotional expression through internal cognitive adjustment. Although it requires cognitive effort initially, deep acting may reduce the prolonged depletion of emotional resources in the long run through positive feedback from its authenticity. When individuals engage more in deep acting, they may experience greater authenticity and less hypocrisy, resulting in a more positive psychological experience that may protect against exhaustion. Therefore, emotional intelligence may directly affect family emotional labor and emotional exhaustion and may also prevent or reduce emotional exhaustion by promoting more adaptive family emotional labor strategies, thus forming a complete “resource (emotional intelligence) – process (family emotional labor strategies) – state (emotional exhaustion) – expectation (childbearing affective reward expectation)” influence chain. Based on this, we propose Hypotheses 3 and 4:

*H3*: Family emotional labor strategies mediate the relationship between emotional intelligence and emotional exhaustion.

*H4*: Family emotional labor strategies and emotional exhaustion play a chain mediating role in the relationship between emotional intelligence and childbearing affective reward expectation.

**Figure 1:**
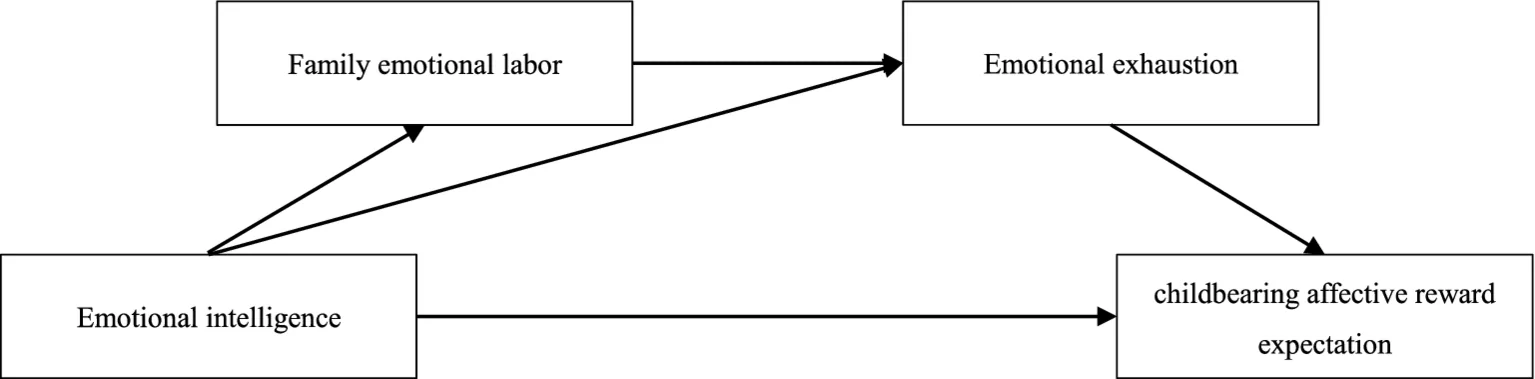
The hypothesized model.

In summary, this study aims to explore the relationship and underlying mechanisms between emotional intelligence and childbearing affective reward expectation. Considering the relationship between emotional intelligence, family emotional labor strategies, emotional exhaustion, and childbearing affective reward expectation, the proposed hypothesized model is shown in Figure 1.

## 2 Materials and methods

### 2.1 Participants

The questionnaire survey data for this study were collected from female healthcare workers at three affiliated hospitals (Hefei, Suzhou, and Fuyang) of a medical college in Anhui Province. Using convenience sampling, female healthcare workers of childbearing age (between 22 and 49 years) were recruited through hospital management departments, such as medical services and nursing departments. To reduce common method bias, we emphasized that this survey was only for academic research purposes. On the front page of the questionnaire, we stated that responses would be anonymous and that all data would be strictly confidential. Considering the large number of questions, we adopted a two-stage questionnaire survey. Each sampling period lasted for 5 days, with a 10-day interval between the two stages. For relatively stable cognitive variables, such as emotional expectations, longitudinal tracking over months or years may better capture dynamic changes and causal relationships; however, given the risk of interrupted data collection, we used a shorter interval between the two measurements. The first letter of the participant’s English surname and the last four digits of their mobile phone number were used to match questionnaires across two stages. In the first stage, data on family emotional labor, emotional intelligence, demographics, and other control variables were collected, and 634 questionnaires were returned. In the second stage, information on marriage and childbearing, such as childbearing affective reward expectation, was collected by following up on the 634 questionnaires from the first stage. First stage questionnaires were considered valid for matching if the respondents participated in the second stage. Respondents lost to follow-up in the second stage were excluded from the sample. Ultimately, 543 valid and matched questionnaires were obtained from the two stages. Additionally, listwise deletion was used to remove cases with missing values in the core variables; responses with excessively short completion times or incorrect answers to logical-check questions were also excluded. Finally, 450 valid responses were retained, corresponding to an effective response rate of 70.98%. Among them, 77 (17.1%) were aged under 30 years, 306 (68.0%) were between 30 and 40 years, and 67 (14.9%) were between 40 and 49 years.

### 2.2 Measurement tools

#### 2.2.1 Emotional intelligence scale

The emotional intelligence scale used in this study was adapted from Wong’s emotional intelligence scale (19). This scale includes four dimensions: self-emotion appraisal, other-emotion appraisal, emotional management, and emotional utilization. A 5-point Likert scale was used for all items, with responses ranging from 1 (completely disagree) to 5 (completely agree). Emotional intelligence was measured using the mean item score, with higher scores indicating greater emotional intelligence. Cronbach’s *α* for the Emotional Intelligence Scale was 0.93. Cronbach’s *α* values for the self-emotion appraisal, other-emotion appraisal, emotional management, and emotional utilization subscales were 0.94, 0.89, 0.95, and 0.92, respectively. Confirmatory factor analysis was used to test the validity of the revised Emotional Intelligence Scale. We retained the original four-dimensional structure and used the modification indices only to establish residual correlations for semantically similar items within the same dimension. The model fit indices were *χ*^2^/df = 3.601, RMSEA = 0.076, GFI = 0.973, NFI = 0.949, CFI = 0.962, TLI = 0.954, SRMR = 0.049. Although the *χ*^2^/df ratio exceeded the critical value of 3, it remained below 5, and the SRMR indicated good model fit, suggesting adequate overall structural validity for subsequent empirical analysis.

#### 2.2.2 Childbearing affective reward expectation

In this study, childbearing affective reward expectation is defined as an individual’s subjective evaluation and belief regarding the positive emotional experience and satisfaction that may arise from future childbearing and parenting before making a childbearing decision. It focuses on the emotional rewards individuals associate with childbearing, such as happiness, love, meaning, and sense of achievement, rather than their attitude or overall willingness toward the fertility behavior itself. In this study, childbearing affective reward expectation was measured using a 5-point Likert scale (1 = very disagree, 2 = disagree, 3 = neutral, 4 = agree, 5 = strongly agree) through the item “bearing children can enrich one’s life and increase happiness.” Higher scores indicate greater childbearing affective reward expectation.

#### 2.2.3 Family emotional labor scale and family emotional labor strategies

According to Hochschild, emotional labor can be divided into two dimensions: surface acting and deep acting (20). The family emotional labor scale was adapted from the emotional labor scale developed by Diefendorff (21). It mainly measures the emotional regulation of the participants in family life to maintain relationships with family members, including parents, siblings, spouses, and children across two dimensions: surface acting and deep acting. The scale uses a 5-point Likert scale, with responses ranging from 1 (never) to 5 (always). Surface and deep acting scores were calculated as the mean value of the corresponding scale items. Higher scores indicate greater levels of surface and deep acting. In this study, the Cronbach’s *α* for the Family Emotional Labor Scale was 0.88, and 0.93 and 0.81 for the surface acting and the deep acting subscales, respectively. This study does not focus on the absolute levels of deep acting or surface acting but rather on the relative advantages of the two strategies of individuals in family scenarios. Therefore, the difference between the standardized deep acting and surface acting was used to measure family emotional labor strategies. Furthermore, the reliability of the difference calculated using the classic formula provided by Bedeian and Armenakis (22, 23) was 0.751, which is within an acceptable range. At the same time, considering the potential reduction in reliability associated with difference scores, we also incorporated deep acting and surface acting as two independent mediating variables in the chain mediation model and examined their respective indirect effects.

#### 2.2.4 Emotional exhaustion scale

The Emotional exhaustion scale, revised by Watkins (24), was employed. It comprises three items, each assessed using a 5-point Likert scale ranging from 1 (completely disagree) to 5 (completely agree). Emotional exhaustion levels were calculated based on the mean scores across all items, with higher values indicating greater emotional exhaustion. In this study, the Cronbach’s *α* coefficient for this scale was 0.86.

#### 2.2.5 Control variables

With reference to the research of Zhi (25), some variables that may affect childbearing affective reward expectation, such as age and personal income, were taken as control variables. At the same time, in the correlation analysis, it was found that the statistical information such as family property and working years was also highly correlated with childbearing affective reward expectation, so it was included in the model as a control variable in the follow-up study.

### 2.3 Data processing and analysis

Data processing and statistical analysis were conducted using SPSS 24.0. The PROCESS macro program was employed to examine chained mediating effects.

## 3 Results

### 3.1 Common method bias test

This study adopted the self-reporting method for measurement. To avoid the influence of common method bias, a two-stage questionnaire survey was adopted, and some questions were scored in reverse. The Harman single-factor method was used in statistics to test the common method deviation. The results showed that there were 7 factors with eigenvalues greater than 1. The variance explained by the first factor was 33.53%, which was less than the critical standard of 40%, indicating that there was no serious common method bias in the study (26).

### 3.2 Descriptive statistics and correlation analysis

Analyses of the mean, standard deviation, and correlation coefficients for emotional intelligence, family emotional labor strategies, emotional exhaustion, and childbearing affective reward expectation were presented in Table 1. Emotional intelligence was significantly positively correlated with childbearing affective reward expectation (*r* = 0.156, *p* < 0.001). Family emotional labor strategies was significantly positively correlated with childbearing affective reward expectation (*r* = 0.109, *p* = 0.020). Emotional exhaustion was significantly negatively correlated with childbearing affective reward expectation (*r* = −0.256, *p* < 0.001).

**Table 1:** Descriptive statistics and correlation analysis of variables.

Variable	*M*	SD	1	2	3	4	5	6	7	8	9	10
1. Age	34.724	5.655	1									
2. Family property	0.831	0.375	0.501^***^	1								
3. Personal income	5.122	1.325	0.195^***^	0.243^***^	1							
4. Working years	3.536	1.025	0.830^***^	0.531^***^	0.252^***^	1						
5. Emotional intelligence	3.730	0.554	−0.048	−0.074	0.061	−0.028	1					
6. Family emotional labor strategies	0.566	0.802	−0.04	−0.137^**^	0.075	−0.055	0.348^***^	1				
7. Surface acting	2.617	0.912	0.037	0.106^*^	−0.045	0.055	−0.102^*^	−0.641^***^	1			
8. Deep acting	3.183	0.733	0.002	−0.019	0.026	0.009	0.253^***^	0.297^***^	0.543^***^	1		
9. Emotional exhaustion	2.237	0.814	−0.064	−0.051	−0.076	−0.055	−0.218^***^	−0.247^***^	0.299^***^	0.102^*^	1	
10. Childbearing affective reward expectation	3.867	0.920	0.148^**^	0.135^**^	0.121^*^	0.130^**^	0.156^***^	0.109^*^	−0.053	0.054	−0.256^***^	1

^*^p < 0.05, ^**^p < 0.01, ^***^p < 0.001, The same applies below; *M*, means; SD, standard deviation; N, 450.

### 3.3 Testing the chain mediation effect

The chain mediating effect test was performed using model 6 in the SPSS PROCESS plug-in. With emotional intelligence as the independent variable, family emotional labor strategies, surface acting and deep acting as the mediating variables M1, M1a, M1b, emotional exhaustion as the mediating variable M2, and childbearing affective reward expectation as the dependent variable, the mediating analysis was carried out, and all variables except the control variables were standardized. The Bootstrap test was set to 5,000 times, and the confidence interval was set to 95%. The regression analysis results were shown in Table 2. The results showed that in the model with family emotional labor strategies and emotional exhaustion as mediating variables, emotional intelligence had a positive effect on childbearing affective reward expectation (*β* = 0.103, *p* = 0.038) and family emotional labor strategies (*β* = 0.359, *p* < 0.001). The regression coefficient of family emotional labor strategies on emotional exhaustion (*β* = −0.150, *p* = 0.003) was significantly negative. Emotional intelligence was negatively correlated with emotional exhaustion (*β* = −0.166, *p* = 0.001). The regression coefficient (*β* = −0.211, *p* < 0.001) of emotional exhaustion on childbearing affective reward expectation was significantly negative. In the model with surface acting and emotional exhaustion as mediating variables, the regression coefficient for surface acting was significantly positive for emotional exhaustion (*β* = 0.287, *p* < 0.001) but not significant for childbearing affective reward expectation (*β* = 0.018, *p* = 0.707); Emotional exhaustion was significantly negatively correlated with childbearing affective reward expectation (*β* = −0.222, *p* < 0.001); Emotional intelligence showed a non-significant correlation with surface acting (*β* = −0.009, *p* = 0.056) but a significant negative correlation with emotional exhaustion (*β* = −0.194, *p* < 0.001); emotional intelligence was significantly positively correlated with childbearing affective reward expectation (*β* = 0.116, *p* = 0.013). In the model with deep acting and emotional exhaustion as mediating variables, emotional intelligence had a significant positive correlation with the regression coefficient for deep acting (*β* = 0.252, *p* < 0.001) and the regression coefficient for childbearing affective reward expectation (*β* = 0.101, *p* = 0.038); deep acting (*β* = 0.169, *p* < 0.001) was significantly positively correlated with emotional exhaustion, but showed no significant correlation with the regression coefficient for childbearing affective reward expectation (*β* = 0.051, *p* = 0.280); emotional exhaustion was significantly negatively correlated with the regression coefficient for childbearing affective reward expectation (*β* = −0.225, *p* < 0.001).

**Table 2:** Regression analysis results of variable relationships in the mediation model.

Regression equation variable		Overall fit index of the equation	Significance of regression coefficients		
Outcome variable	Predictor variable	*R* ^2^	*Βeta* (95%CI)	*t*	*p*
Model 1: family emotional labor strategies model					
Family emotional labor strategies	Emotional intelligence	0.157	0.359 (0.259–0.423)	8.175	<0.001
Emotional exhaustion	Emotional intelligence	0.075	−0.166 (−0.259 to −0.068)	−3.367	0.001
	Family emotional labor strategies		−0.150 (−0.257 to −0.054)	−3.016	0.003
Childbearing affective reward expectation	Emotional intelligence	0.106	0.103 (0.006–0.196)	2.086	0.038
	Family emotional labor strategies		0.038 (−0.061 to 0.141)	0.777	0.437
	Emotional exhaustion		−0.211 (−0.303 to −0.120)	−4.521	<0.001
Model 2: surface acting model					
Surface Acting	Emotional intelligence	0.026	−0.090 (−0.182 to 0.002)	−1.915	0.056
Emotional exhaustion	Emotional intelligence	0.136	−0.194 (−0.277 to −0.105)	−4.348	<0.001
	Surface acting		0.287 (0.197–0.371)	6.412	<0.001
Childbearing affective reward expectation	Emotional intelligence	0.105	0.116 (0.024–0.204)	2.492	0.013
	Surface acting		0.018 (−0.075 to 0.111)	0.376	0.707
	Emotional exhaustion		−0.222 (−0.317 to −0.127)	−4.583	<0.001
Model 3: deep acting model					
Deep acting	Emotional intelligence	0.065	0.252 (0.161–0.342)	5.464	<0.001
Emotional exhaustion	Emotional intelligence	0.097	−0.263 (−0.350 to −0.167)	−5.551	<0.001
	Deep acting		0.169 (0.076–0.259)	3.596	<0.001
childbearing affective reward expectation	Emotional intelligence	0.107	0.101 (0.005–0.193)	2.079	0.038
	Deep acting		0.051 (−0.041 to 0.142)	1.081	0.280
	Emotional exhaustion		−0.225 (−0.317 to −0.133)	−4.797	<0.001

**Figure 2:**
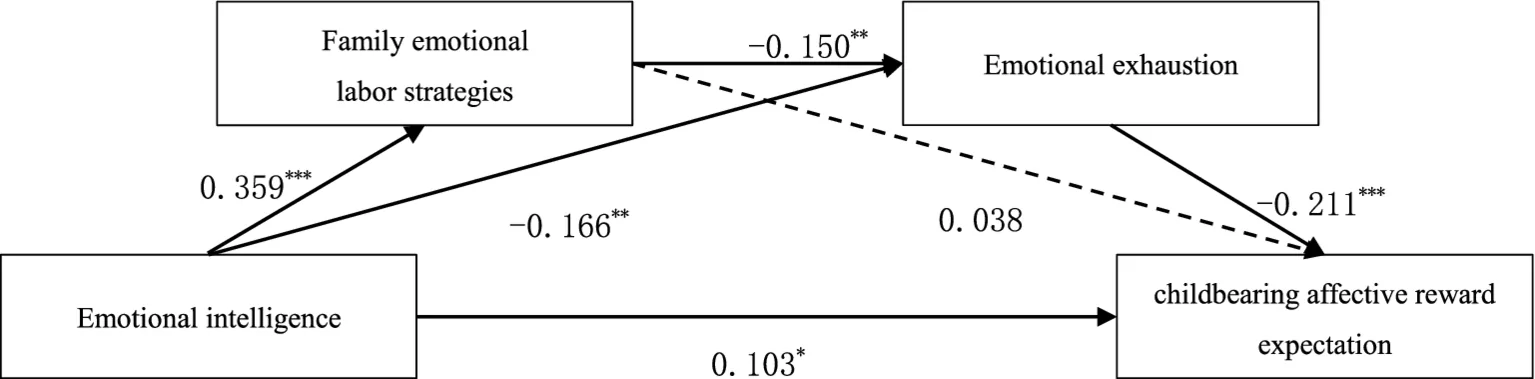
Path analysis diagram (Model 1: family emotional labor strategies model). Values represent standardized regression coefficients.

**Figure 3:**
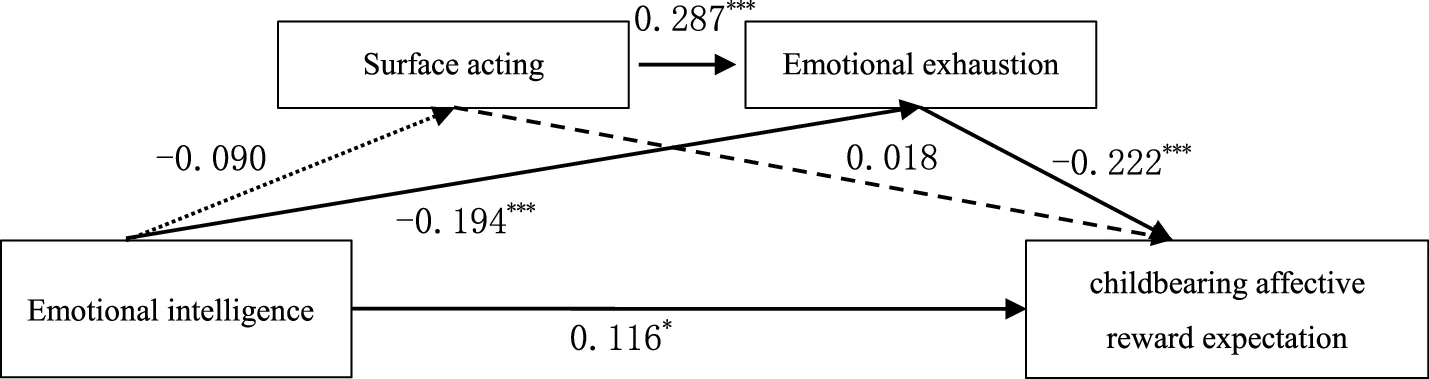
Path analysis diagram (Model 2: surface acting model). Values represent standardized regression coefficients.

**Figure 4:**
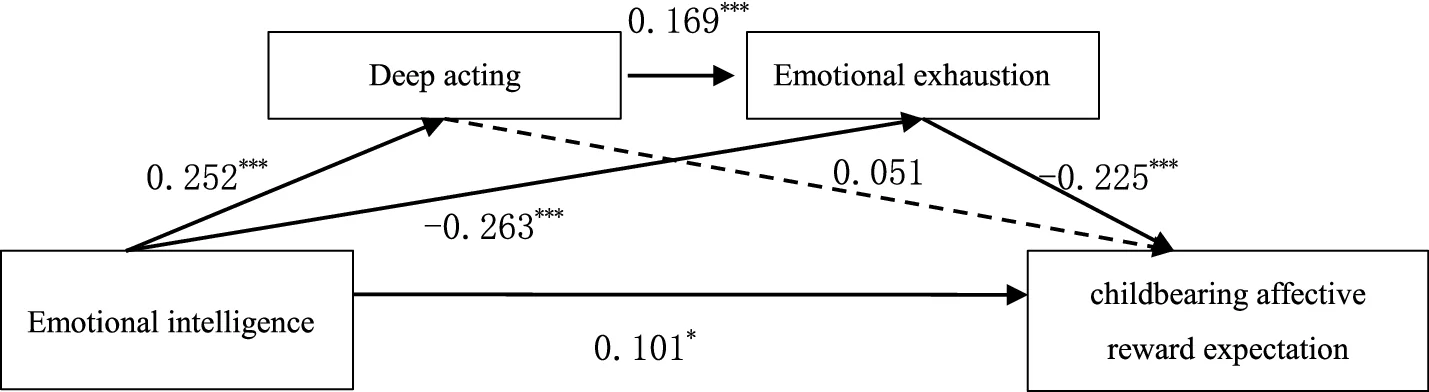
Path analysis diagram (Model 3: deep acting model). Values represent standardized regression coefficients.

The results of mediating effect analysis showed that there was a direct correlation between emotional intelligence and childbearing affective reward expectation, with a direct effect of 0.103, 95% CI (0.006–0.196). Emotional intelligence was indirectly related to childbearing affective reward expectation through the chain mediating path of family emotional labor strategies and emotional exhaustion, with a total indirect effect of 0.060, 95% CI (0.015–0.104). Specifically, the mediating effect was mainly composed of two indirect paths: the indirect effect generated by the path of emotional intelligence → emotional exhaustion → childbearing affective reward expectation was 0.035, 95% CI (0.009–0.062); the indirect effect generated by the path of emotional intelligence → family emotional labor strategies → emotional exhaustion → childbearing affective reward expectation was 0.011, 95% CI (0.002–0.026). The Bootstrap 95% CI of the two paths did not contain 0, indicating that the two indirect effects were significant. However, the indirect effect generated by the path of the emotional intelligence → family emotional labor strategies → childbearing affective reward expectation was 0.014 and the Bootstrap 95% CI contained 0, indicating that the separate mediating effect of family emotional labor strategies on the relationship between emotional intelligence and childbearing affective reward expectation was not significant. The results were shown in Table 3 and Figures 2–4.

**Table 3:** Bootstrap analysis of chain mediating effect.

Effect type	Effect	Boot SE	Boot LLCI	Boot ULCI	Proportion of total effect (%)
Model 1: family emotional labor strategies model					
Indirect 1: EI → FELS → CARE	0.014	0.018	−0.022	0.051	8.59%
Indirect 2: EI → EE → CARE	0.035	0.014	0.009	0.062	21.47%
Indirect 3: EI → FELS → EE → CARE	0.011	0.006	0.002	0.026	6.75%
Indirect effect	0.060	0.023	0.015	0.104	36.81%
Direct effect	0.103	0.048	0.006	0.196	63.19%
Total effect	0.163	0.046	0.071	0.250	100.00%
Model 2: surface acting model					
Indirect 1: EI → SA → CARE	−0.002	0.005	−0.014	0.009	−1.23%
Indirect 2: EI → EE → CARE	0.043	0.015	0.017	0.075	26.38%
Indirect 3: EI → SA → EE → CARE	0.006	0.004	−0.001	0.015	3.68%
Indirect effect	0.047	0.016	0.017	0.082	28.83%
Direct effect	0.116	0.046	0.024	0.204	71.17%
Total effect	0.163	0.046	0.071	0.250	100%
Model 3: deep acting model					
Indirect 1: EI → DA → CARE	0.013	0.014	−0.013	0.043	7.98%
Indirect 2: EI → EE → CARE	0.059	0.018	0.026	0.095	36.20%
Indirect 3: EI → DA → EE → CARE	−0.010	0.005	−0.021	−0.003	−6.13%
Indirect effect	0.062	0.022	0.019	0.105	38.04%
Direct effect	0.101	0.048	0.005	0.193	61.96%
Total effect	0.163	0.046	0.071	0.250	100%

EI, Emotional intelligence; FELS, Family emotional labor strategies; SA, Surface acting; DA, Deep acting; EE, Emotional exhaustion; CARE, childbearing affective reward expectation. Boot SE, bootstrapping standard error; Boot LLCI, bootstrapping lower limit confidence interval; Boot ULCI, bootstrapping upper limit confidence interval; Effect, standardized regression coefficient.

## 4 Discussion

First, correlation analysis showed a significant positive correlation between emotional intelligence and childbearing affective reward expectation among female healthcare workers of childbearing age (*r* = 0.156, *p* < 0.001). Further multiple regression analysis showed a significant positive association between emotional intelligence and childbearing affective reward expectation (*β* = 0.103, *p* = 0.038). This finding is consistent with previous research suggesting that emotional intelligence can promote emotional regulation (27, 28). Female healthcare workers of childbearing age with higher emotional intelligence may be able to identify their own emotional problems in the context of family emotional labor and improve their childbearing affective reward expectation by adjusting their emotional strategies in a timely manner.

Second, this study initially found that emotional exhaustion mediates the relationship between emotional intelligence and childbearing affective reward expectation. The higher the emotional intelligence of female healthcare workers, the stronger their ability to evaluate and use emotions. Individuals with high emotional intelligence have a stronger ability to recognize and regulate emotions. In high-pressure situations, they use adaptive strategies for cognitive reappraisal and emotional repair, rather than depression or evasion, thereby reducing the psychological cost of emotional labor (29). Existing studies have found that female healthcare workers with high emotional intelligence can better manage family-work conflicts and reduce psychological distress and anxiety than individuals with low emotional intelligence (30). Therefore, female healthcare workers with high emotional intelligence are calmer in the face of emotional shocks in family settings, conserve psychological resources, and alleviate the perceived threat associated with fertility, that is, resource loss, thereby maintaining positive childbearing affective reward expectation.

Considering potential limitations, such as the reduced reliability associated with measuring family emotional labor strategies using a difference score, we simultaneously incorporated deep acting and surface acting as two independent mediating variables into the chain mediating model to examine their respective indirect effects. This strategy avoids the problem of reduced reliability associated with difference scores and can more accurately estimate the independent contribution of each strategy. Figures 3, 4 showed that emotional intelligence has a significant positive effect on deep acting (*β* = 0.252, *p* < 0.001), whereas its effect on surface acting was not statistically significant (*β* = −0.009, *p* = 0.056). Both deep and surface acting were significantly and positively correlated with emotional exhaustion. However, the regression coefficient (*β* = 0.287, *p* < 0.001) of surface acting on emotional exhaustion was greater than that of deep acting on emotional exhaustion (*β* = 0.169, *p* < 0.001), which also explains the significant negative effect of family emotional labor strategies on emotional exhaustion. This is mainly because emotional intelligence promotes deep acting among female healthcare workers of childbearing age, and deep acting has a smaller effect on emotional exhaustion. These findings are logically consistent. The relative dominance of deep acting helps alleviate emotional exhaustion, indicating that the research conclusion does not rely on a specific method for calculating the difference score. Therefore, increasing the proportion of deep acting in family emotional labor and enabling women to genuinely recognize the positive feedback from emotional labor in family settings may warrant consideration in subsequent policies.

## 5 Conclusion

The purpose of this study is to examine the relationship between emotional intelligence and childbearing affective reward expectation, and the mediating role of family emotional labor strategies and emotional exhaustion between them. The study supports the following hypotheses: First, there is a significant positive correlation between emotional intelligence and childbearing affective reward expectation; second, emotional exhaustion plays a mediating role between emotional intelligence and childbearing affective reward expectation; third, family emotional labor strategies and emotional exhaustion play a chain mediating role between emotional intelligence and childbearing affective reward expectation.

## 6 Limitations and future research prospects

This study has the following limitations. First, the scope of variable measurement was limited. The dependent variable, childbearing affective reward expectation, was measured using a single item. Although existing research suggests that using a single indicator for measuring outcome variables such as happiness (31), this approach limits the variable’s measurement reliability and validity to a certain extent and reduces the robustness of the regression analysis and mediating effect test results. Future studies should use multi-item and multidimensional scales to measure this variable more comprehensively and reliably. Second, there are limitations related to sample representativeness. The sample consisted of female healthcare workers from three hospitals in Anhui Province. Their work stress and emotional labor intensity may be higher than those of the general population, and the sample representativeness is therefore limited. The generalizability of the study’s conclusions should be examined using a broader sample in future research. Third, the time interval between the two surveys in this study was too short, limiting the ability to draw causal inferences. Moreover, all data were collected using self-reported questionnaires, which may have introduced measurement bias. Future research could use longitudinal follow-up surveys combined with objective behavioral indicators, multi-party evaluations, and other multi-measurement methods to further explore the mechanisms underlying the relationships among the variables.

## Data Availability

The raw data supporting the conclusions of this article will be made available by the authors, without undue reservation.
